# Same-Day Versus Next-Day Discharge After Laparoscopic Appendectomy for Uncomplicated Appendicitis in Children: A Propensity Score-Matched Cost Analysis

**DOI:** 10.3390/children13091136

**Published:** 2026-08-25

**Authors:** Lucas Moratilla-Lapeña, Jose Luis Encinas, Esmeralda Kuan, Francisco Hernández

**Affiliations:** 1 Pediatric Surgery Department, Hospital Universitario La Paz, 28046 Madrid, Spainfhernandezo@salud.madrid.org (F.H.); 2 Pediatric Surgery Department, Complejo Hospitalario Universitario de Toledo, 45007 Toledo, Spain; 3 La Paz Research Institute (Idipaz), Hospital Universitario La Paz, 28046 Madrid, Spain; 4 European Reference Network on Transplantation in Children (TransplantChild ERN), 28046 Madrid, Spain

**Keywords:** appendicitis, ERAS, pediatric surgery, healthcare costs

## Abstract

**Highlights:**

**What are the main findings?**

- Propensity score-matched analysis found that same-day discharge after laparoscopic appendectomy for uncomplicated pediatric appendicitis reduced total healthcare costs by approximately €320 per patient, driven almost entirely by lower admission-related expenditure.
- Complication rates were low and comparable between same-day and next-day discharge groups, with no signal of increased risk associated with early discharge.

**What are the implications of the main findings?**

- Same-day discharge represents a feasible, cost-effective component of ERAS perioperative pathways for pediatric uncomplicated appendicitis, when applied with strict patient selection criteria.
- Larger, multicenter studies with adequate statistical power for complication outcomes are needed to confirm the safety of this strategy before broader clinical adoption.

**Abstract:**

Background: Same-day discharge after laparoscopic appendectomy for uncomplicated appendicitis has been proposed as a strategy to reduce hospital costs without compromising safety, but evidence in pediatric patients remains limited. Methods: We conducted a retrospective study of pediatric patients undergoing laparoscopic appendectomy for uncomplicated appendicitis, comparing same-day and next-day discharge. Baseline differences were addressed using 1:2 propensity score matching. Costs were analyzed using linear regression with cluster-robust standard errors, and inverse probability of treatment weighting was used as a sensitivity analysis. Results: Among 102 patients (37 same-day discharge and 65 delayed discharge), 28 same-day discharge patients were matched to 36 delayed-discharge controls. Same-day discharge was associated with a mean reduction in total cost of €319.9 (95% CI: −€413.6 to −€226.2; *p* < 0.0001), mainly explained by lower admission-related costs, with no significant difference in outpatient consultation costs. Sensitivity analysis using inverse probability weighting in the full cohort showed a similar estimate (−€311.7; 95% CI: −€421.1 to −€202.3; *p* < 0.0001). Complications were rare and similar in both groups (1/28 vs. 1/36), which limited formal comparison of complication risk. Conclusions: Same-day discharge after laparoscopic appendectomy for uncomplicated appendicitis was associated with lower healthcare costs, largely due to reduced admission-related costs, with no apparent signal of increased complications.

## 1. Introduction

Acute appendicitis is the most common cause of acute abdomen in the pediatric population [1]. Despite its high prevalence, no worldwide consensus exists regarding its therapeutic management [2,3,4]. Several classification systems have been proposed to stratify appendicitis according to disease severity, with the most widely used being the simple versus complicated appendicitis dichotomy, although new standards based on intraoperative findings have recently been introduced to further define the different grades of disease complexity [5,6,7].

There is currently a broad consensus within the scientific community regarding the treatment of acute appendicitis in children, with evidence showing that antibiotic therapy beyond the single preoperative dose is not required [8]. This finding implies that hospital admission beyond postoperative recovery is unnecessary. In order to optimize this recovery period, Enhanced Recovery After Surgery (ERAS) protocols were developed, initially implemented in adults and subsequently extended to the pediatric population, with highly satisfactory outcomes and no increase in complication rates compared with more conservative protocols [9,10,11,12]. In recent years, the implementation of ERAS protocols has progressively expanded to multiple procedures within pediatric surgery. Our department has developed a specific protocol for the management of acute appendicitis. In complicated cases, a five-day hospital stay is established, determined mainly by the need for intravenous antibiotic therapy. In contrast, in uncomplicated appendicitis, which does not require postoperative antibiotic therapy, hospital discharge is based on postoperative recovery and is left to the discretion of the surgeon.

It is worth noting that, in most cases of uncomplicated acute appendicitis, the surgical procedure lasts less than 45 min, comparable to other ambulatory laparoscopic procedures. In the adult population, several studies have demonstrated that optimizing ERAS protocols with same-day discharge after surgery is a feasible, safe, and cost-effective strategy that does not increase complication rates [13,14,15,16] and, furthermore, improves satisfaction among both patients and caregivers [17]. The pediatric literature on the implementation of ERAS protocols with same-day discharge remains scarce; however, the studies published to date show outcomes comparable to those described in adults, with promising results [18,19,20].

For these reasons, the primary objectives of the present study are: to evaluate whether a higher complication rate exists among pediatric patients undergoing surgery for uncomplicated acute appendicitis who are discharged on the same day as surgery, compared with those discharged the following day, and to assess the economic impact of early discharge in this population.

## 2. Methods

### 2.1. Study Design

A retrospective observational study was conducted including pediatric patients treated at the Department of Pediatric Surgery of the Complejo Hospitalario Universitario de Toledo who underwent appendectomy for acute appendicitis between 2020 and 2025.

### 2.2. Eligibility Criteria and Group Allocation

Patients were eligible for inclusion if they met all of the following criteria: age < 14 years, diagnosis of uncomplicated acute appendicitis, treatment by laparoscopic approach, and surgery performed during the morning shift. Those who did not meet all these criteria were excluded. Patients were then classified into two groups according to discharge timing: same-day discharge and those discharged on a later date.

Only patients operated on during the morning shift were eligible, to ensure sufficient time for postoperative observation and clinical reassessment prior to a potential same-day discharge. All appendectomies were performed by an attending pediatric surgeon. A laparoscopic approach was required for inclusion because it constitutes the standard surgical technique for uncomplicated appendicitis within our department’s ERAS protocol and was specified as an eligibility criterion to ensure a homogeneous surgical technique across the study. The choice between multiportal and transumbilical laparoscopic-assisted approach was based on the individual surgeon’s preference.

The decision to discharge a patient on the day of surgery versus on a subsequent day was made at the discretion of the treating surgeon based on overall postoperative clinical recovery; our department does not apply a standardized, itemized discharge checklist.

### 2.3. Variables

The following variables were recorded for each patient: demographic (age, weight, and sex), clinical (duration of symptoms before admission, presence of fever, vomiting, and diarrhea), analytics (leukocyte, neutrophil, lymphocyte and monocyte count, C-reactive protein, and prothrombin time), surgical approach (multiportal or transumbilical laparoscopic-assisted surgical approach), post-discharge emergency department visit, presence and type of complication (Clavien–Madadi classification [21]) and admission-related and outpatient consultation costs.

### 2.4. Cost Analysis

Admission-related costs and complications were estimated according to the reference values established by the Resolution of 6 April 2010 of the Management Board of SESCAM (Servicio de Salud de Castilla-La Mancha). According to this framework, the cost of one day of hospital admission was €491.16 when an overnight stay occurred, whereas cases without an overnight stay were assigned 50% of this amount. The cost of one emergency department visit was €187.61, and each additional follow-up visit related to complications was valued at €72.36. Costs related to the initial emergency surgical procedure were not included in the analysis because they were considered equivalent in both groups.

### 2.5. Statistical Analysis

Continuous variables were summarized as median and standard deviation (SD) or median and interquartile range (IQR), according to data distribution, and categorical variables as absolute and relative frequencies. Baseline characteristics were compared between groups using Student’s *t*-test or the Wilcoxon rank-sum test for continuous variables, and the chi-square test or Fisher’s exact test for categorical variables, as appropriate.

To reduce confounding due to baseline differences between groups, a propensity score (PS) analysis was performed [22,23]. The PS was estimated using a logistic regression model and defined as the probability of same-day discharge conditional on prespecified baseline covariates. Covariates were selected a priori according to their potential association with both discharge status and study outcomes. The model included age, sex, weight, leukocyte count, C-reactive protein, duration of symptoms before admission, and surgical approach. This last covariate was included because the choice between transumbilical-assisted and multiportal approach was largely determined by individual surgeon preference rather than by patient-specific clinical criteria, and was therefore considered a potential source of confounding.

Patients were matched in a 1:2 ratio using nearest-neighbor matching without replacement, with estimation of the average treatment effect on the treated. A caliper of 0.2 standard deviations of the logit of the propensity score was used. Covariate balance before and after matching was assessed using standardized mean differences (SMDs), considering values < 0.25 as indicative of acceptable balance. Balance was also examined graphically.

Outcomes were analyzed in the matched cohort. For total cost, a linear regression model was fitted with cluster-robust standard errors at the matched-set level. Because the number of postoperative complications was low, this outcome was analyzed descriptively, and no multivariable model was fitted. As a sensitivity analysis, the treatment effect on total cost was additionally estimated using inverse probability of treatment weighting (IPTW), applied to the full, unmatched cohort. Stabilized weights were calculated as the ratio of the marginal probability of the observed treatment to the propensity score, using the same covariates as the primary propensity score model. A linear regression model was fitted to the IPTW-weighted full sample, with heteroskedasticity-consistent standard errors to account for the variability introduced by the weights. This approach provided an estimate of the average treatment effect (ATE) in the full population, complementing the average treatment effect on the treated (ATT) estimated through matching, and served to assess whether the estimated cost reduction was consistent across different propensity score methods.

All analyses were performed using R software (version 4.5.3; R Foundation for Statistical Computing, Vienna, Austria). PS estimation, matching, and balance assessment were performed using the *MatchIt* and *cobalt* packages; cluster-robust and heteroskedasticity-consistent variance estimation using *sandwich* and *lmtest*; and descriptive tables using *gtsummary*. All tests were two-sided, and *p* < 0.05 was considered statistically significant.

## 3. Results

### 3.1. Study Population

A total of 102 pediatric patients who underwent laparoscopic appendectomy for uncomplicated acute appendicitis between 2020 and 2025 met the inclusion criteria and were included in the analysis. Of these, 37 patients (36.3%) were discharged on the same day as surgery, and 65 patients (63.7%) were discharged on a subsequent day.

Table 1 summarizes the baseline demographic, clinical, and laboratory characteristics of both groups prior to matching. Several statistically significant differences were observed between groups. Patients discharged on the same day had lower body weight (*p = 0.006*), lower leukocyte count (*p = 0.007*), and lower neutrophil count *(p = 0.007*) compared to those with delayed discharge. A transumbilical laparoscopic-assisted surgical approach (TULAA) was more frequent in the same-day discharge group (92% vs. 74%, *p = 0.037*), with surgical time being shorter in this group (*p < 0.001*).

### 3.2. Propensity Score Matching and Covariate Balance

Of the 37 patients discharged on the same day, 28 (75.7%) were successfully matched to 36 next-day discharge controls. The remaining 9 same-day discharge patients could not be matched within the specified caliper and were excluded from the matched analysis. Although a 1:2 matching ratio was specified, not all treated patients could be matched to two eligible controls within the specified caliper; some were matched to only one control, resulting in an overall control-to-treated ratio of approximately 1.3:1 rather than 2:1.

**Figure 1:**
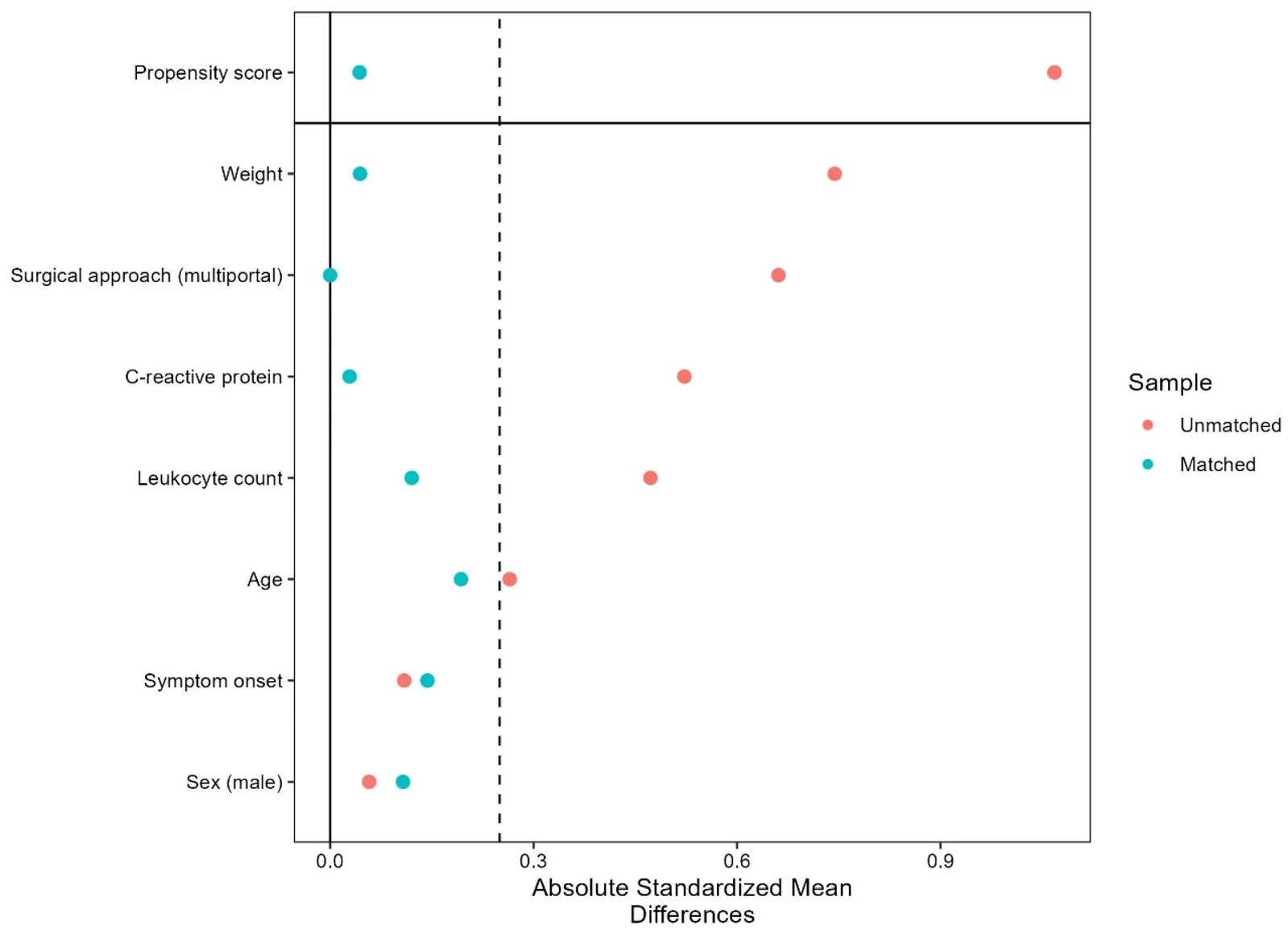
**Covariate balance before and after 1:2 propensity score matching.** Absolute standardized mean differences (SMDs) are shown for each covariate included in the propensity score model, comparing the unmatched sample and the matched sample. The dashed line indicates the conventional balance threshold of SMD = 0.25.

Covariate balance improved substantially after matching (Figure 1). SMDs decreased for all included covariates, with values below 0.2 for every variable and below 0.1 for the majority. Table 2 presents the baseline characteristics of the matched sample. After PS matching, no statistically significant differences remained between groups for any of the covariates included in the propensity score model, confirming adequate covariate balance. Only surgical time remained significantly different between groups after matching (30 [20–40] vs. 35 [30–43] min, *p = 0.002*).

### 3.3. Postoperative Complications and Healthcare Utilization After Surgery

In the matched sample, one complication was observed in each group (1/28 [3.6%] in the same-day discharge group and 1/36 [2.8%] in the delayed-discharge group). Both complications were surgical site infections, classified as Clavien–Madadi grade IB in both cases. Each complication resulted in one emergency department visit after discharge. Subsequent outpatient follow-up differed between groups: the patient in the same-day discharge group required four additional follow-up visits, compared with two additional visits for the patient in the delayed-discharge group. It is worth noting that these were the only two complications observed across the entire cohort of 102 patients, including those excluded from the matched analysis.

### 3.4. Cost Analysis Results

In the matched sample, same-day discharge reduced mean total cost by €319.9 compared with delayed discharge (95% CI: €226.2 to €413.6; *p < 0.0001*). When the total cost was broken down into its main components, the difference was explained almost entirely by lower admission-related costs. Mean admission cost was €333.3 lower in the same-day discharge group (95% CI: €264.9 to €401.7; *p < 0.0001*). By contrast, outpatient consultation costs were slightly higher, but the difference was not statistically significant (+€13.4; 95% CI: −€47.9 to €74.7; *p = 0.66*). Overall, the lower total cost observed with same-day discharge was driven by savings in hospital admission, whereas outpatient follow-up costs were broadly similar between groups.

IPTW as a sensitivity analysis was applied to the full unmatched cohort (N = 102). This approach estimated an ATE of −€311.7 (95% CI: −€421.1 to −€202.3; *p < 0.0001*), which was very similar to the ATT estimated with PS matching (−€319.9; 95% CI: −€413.6 to −€226.2; *p < 0.0001*). The similarity between these estimates suggests that the lower cost associated with same-day discharge was not materially influenced by the propensity score method used.

## 4. Discussion

This PS-matched study of pediatric patients undergoing laparoscopic appendectomy for uncomplicated appendicitis found that same-day discharge was associated with a significant reduction in total healthcare costs compared with next-day discharge. This difference was driven primarily by lower admission-related costs and remained consistent across adjusted and sensitivity analyses using two different propensity score approaches. Although postoperative complications were infrequent and appeared similar between groups, the small number of events limits any firm conclusion regarding comparative safety.

Same-day discharge after laparoscopic appendectomy for uncomplicated appendicitis has been increasingly adopted in adult surgical practice as part of broader ERAS-based perioperative pathways, with several studies demonstrating that this approach is both feasible and safe, without an accompanying increase in postoperative complications [13,14,15,16]. Nonetheless, pediatric evidence on same-day discharge has historically lagged behind the adult literature, though the number of supporting studies has grown considerably in recent years. According to this growing body of literature, same-day discharge after laparoscopic appendectomy for uncomplicated appendicitis in children appears to be an increasingly adopted strategy that aligns with current efforts to improve efficiency and value in pediatric surgical care, while avoiding unnecessary overnight hospitalization [18,24,25]. This trend is supported by large contemporary datasets showing that same-day discharge rates rose from 33.3% to 52.5% in NSQIP-P between 2017 and 2021 [26] and by institutional pathway studies in which structured protocols doubled the same-day discharge rate [19,27]. Also, this expansion has been accompanied by a consistent safety signal, with no detected increase in readmissions, emergency department visits, wound complications, or overall postoperative morbidity compared with next-day or short-stay discharge, including in multicenter registry data, prospective cohorts, systematic reviews, and meta-analyses [15,19,20,24,25,26].

Our results are in line with previously published studies, with a similarly low rate of complications. Only two complications were observed across the full cohort, and neither required hospital readmission, reinforcing the safety of same-day discharge in the pediatric population.

Beyond clinical safety, patient and caregiver acceptability is an important consideration when evaluating same-day discharge protocols. A recent study by Cruz-Centeno et al. demonstrated that caregiver satisfaction with same-day discharge following laparoscopic appendectomy for uncomplicated appendicitis in children is high (91.5%), with most caregivers reporting adequate postoperative pain control using nonopioid analgesics, suggesting that same-day discharge is both safe and well accepted [17]. This idea was previously published by Alkhoury et al., with similar reported results, with low rates of complications (surgical site infections, 2%) and low rates of readmissions (3%) [28]. This high caregiver satisfaction has also been noted in other studies [18].

Despite this favorable safety and acceptability profile, implementation of same-day discharge protocols in clinical practice is not without challenges. Buss et al. in their study identified potential barriers to implementing same-day discharge in the pediatric population, which depends less on medical safety than on modifiable operational barriers such as operative timing, protocol compliance, travel distance, and social context [29].

Finally, adoption of a protocolized same-day discharge pathway after laparoscopic appendectomy for uncomplicated appendicitis in children is consistently associated with lower hospital costs, largely through shortening postoperative stay and avoiding unnecessary overnight admission. Recent meta-analyses and systematic reviews estimate that these protocols could reduce costs by a mean difference of $2587.4 per patient (95% CI: −$4628.3, −$546.6) [20], while other reports report reductions that range from $323 to $4111 per patient [15]. This pattern is reproduced in single-center pediatric cohorts, with lower median or mean costs reported for same-day discharge versus overnight observation, including $29,150 vs. $34,827 [25], $10,551 vs. $12,691 [30], and $29,195 vs. $33,703 [31]. Additional institutional estimates outside the United States similarly suggest meaningful aggregate savings, with one prospective study estimating at least €146.10 saved per patient and approximately €23,372.62 annually after a one-day discharge protocol [18].

Our findings are consistent with this body of evidence, showing a mean cost reduction of €319.9 per patient (95% CI −€413.6 to −€226.2) associated with same-day discharge. The stability of our estimate across two independent propensity score approaches therefore supports same-day discharge as a reproducible cost-saving strategy in children with uncomplicated appendicitis. Although the absolute savings vary across institutions and health systems, the direction of effect is consistent, with systematic reviews, cohort studies, and pathway-based quality improvement reports all showing reduced charges, lower cost of stay, or fewer hospital days after adoption of same-day discharge.

Taken together, these findings suggest that a same-day discharge protocol for uncomplicated appendicitis enhances the value of care, reducing per-patient expenditure while freeing hospital capacity, without any detectable increase in readmissions, emergency visits, or postoperative complications among appropriately selected children.

### Limitations

This study has several limitations. First, although 1:2 propensity score matching allowed retention of a larger matched sample, one-to-many matching may yield fewer close matches than 1:1 matching and therefore may increase the risk of residual bias. In addition, the prespecified 1:2 ratio was not fully achieved because some treated patients could only be matched to one control within the caliper, resulting in a lower effective matching ratio.

Second, as with any PS analysis, adjustment was limited to measured covariates. Unmeasured factors that may have influenced both discharge decisions and outcomes, such as surgeon preference, bed availability, distance to the hospital, or family support, could not be assessed and may have resulted in residual confounding. Consistent with this, surgical time remained significantly longer in the next-day discharge group even after matching (median 35 vs. 30 min, *p* = 0.002); although statistically significant, this 5 min difference was modest in absolute terms and unlikely, on its own, to be clinically meaningful or substantial enough to influence the decision to discharge a patient on the same day versus the next day. It may nonetheless reflect unmeasured intraoperative complexity acting as one such residual confounder.

Third, the term “laparoscopic appendectomy” in this study encompasses both conventional multiport laparoscopic appendectomy and TULAA, a laparoscopic-assisted technique in which appendiceal resection is completed extracorporeally. Although both approaches share laparoscopic exploration and mobilization, this heterogeneity in surgical technique should be considered when interpreting and generalizing our findings.

Finally, the retrospective single-center design and the limited sample size, particularly the very low number of complication events, precluded a robust multivariable analysis of postoperative complications and may limit the generalizability of the findings to other settings.

## 5. Conclusions

Same-day discharge after laparoscopic appendectomy for uncomplicated pediatric appendicitis was associated with a reduction in healthcare costs of approximately €320 per patient, driven primarily by avoidance of overnight admission, without a detectable increase in complications. However, given the low complication event rate, larger multicenter studies are needed to define the safety of this strategy with greater precision.

## Figures and Tables

**Table 1:** Baseline characteristics by discharge group.

	Next-Day Discharge N = 65 ^1^	Same-Day Discharge N = 37 ^1^	*p*-Value ^2^
Age (years)	10.33 (8.50, 12.33)	9.33 (8.42, 11.25)	0.15
Sex			0.8
Female	28 (43%)	17 (46%)	
Male	37 (57%)	20 (54%)	
Weight (kg)	42 (32, 50)	31 (26, 40)	0.006
Leukocyte count	16,000 (13,100, 18,100)	13,200 (10,000, 16,200)	0.007
Neutrophil count	12,800 (9800, 14,800)	10,500 (7100, 12,200)	0.007
Lymphocyte count	1900 (1200, 2600)	1900 (1340, 2700)	0.7
Monocyte count	900 (800, 1200)	900 (700, 1100)	0.059
C-reactive protein (mg/L)	8 (1, 16)	7 (1, 15)	0.6
Prothrombin time	80 (74, 87)	81 (75, 90)	0.6
Symptom onset (hours)	12 (6, 24)	18 (12, 24)	0.4
Fever	5 (7.7%)	4 (11%)	0.7
Vomiting	28 (43%)	15 (41%)	0.8
Diarrhea	3 (4.6%)	6 (16%)	0.069
Surgical approach			0.037
TULAA	48 (74%)	34 (92%)	
Multiportal	17 (26%)	3 (8.1%)	
Surgical time (min)	35 (30, 40)	30 (20, 35)	<0.001

^1^ Median (Q1, Q3); N (%). ^2^ Wilcoxon rank sum test; Fisher’s exact test.

**Table 2:** Baseline characteristics of the propensity score-matched sample, stratified by timing of hospital discharge (same-day vs. next-day discharge).

	Next-Day Discharge N = 36 ^1^	Same-Day Discharge N = 28 ^1^	*p*-Value ^2^
Age (years)	9.96 (8.17, 11.75)	9.42 (8.67, 11.42)	>0.9
Sex			0.6
Female	15 (42%)	14 (50%)	
Male	21 (58%)	14 (50%)	
Weight (kg)	37 (26, 45)	34 (28, 46)	0.7
Leukocyte count	15,700 (12,150, 18,000)	13,550 (10,300, 16,555)	0.2
Neutrophil count	12,850 (9350, 14,450)	10,850 (7300, 13,050)	0.2
Lymphocyte count	1850 (1200, 2400)	1800 (1250, 2650)	0.8
Monocyte count	900 (700, 1200)	950 (750, 1100)	0.7
C-reactive protein (mg/L)	3 (1, 17)	7 (1, 16)	0.6
Prothrombin time	79 (74, 90)	82 (75, 89)	0.7
Symptom onset (hours)	12 (6, 24)	19 (12, 24)	0.11
Fever	2 (5.6%)	3 (11%)	0.6
Vomiting	17 (47%)	13 (46%)	>0.9
Diarrhea	2 (5.6%)	5 (18%)	0.2
Surgical approach			>0.9
TULAA	31 (86%)	25 (89%)	
Multiportal	5 (14%)	3 (11%)	
Surgical time (min)	35 (30, 43)	30 (20, 40)	0.002

^1^ Median (Q1, Q3); N (%). ^2^ Wilcoxon rank sum test; Fisher’s exact test.

## Data Availability

The raw data supporting the conclusions of this article will be made available by the authors on request.
